# Supplementary material for: Recurrent connections facilitate occluded object recognition by explaining-away
Source: Nat Commun. 2026 Jan 31;17:2225. doi: 10.1038/s41467-026-68806-5 (PMC12963382; doi:10.1038/s41467-026-68806-5)
Supplement: Supplementary file 2 — Reporting Summary [file 41467_2026_68806_MOESM2_ESM.pdf]

nature portfolio

Corresponding author(s): Shaul Druckmann

Last updated by author(s): Apr 7, 2025

Reporting Summary

Nature Portfolio wishes to improve the reproducibility of the work that we publish. This form provides structure for consistency and transparency in reporting. For further information on Nature Portfolio policies, see our Editorial Policies and the Editorial Policy Checklist.

Statistics

For all statistical analyses, confirm that the following items are present in the figure legend, table legend, main text, or Methods section.

n/a	Confirmed	
☐	☒	The exact sample size (*n*) for each experimental group/condition, given as a discrete number and unit of measurement
☐	☒	A statement on whether measurements were taken from distinct samples or whether the same sample was measured repeatedly
☐	☒	The statistical test(s) used AND whether they are one- or two-sided<br>*Only common tests should be described solely by name; describe more complex techniques in the Methods section.*
☒	☐	A description of all covariates tested
☒	☐	A description of any assumptions or corrections, such as tests of normality and adjustment for multiple comparisons
☐	☒	A full description of the statistical parameters including central tendency (e.g. means) or other basic estimates (e.g. regression coefficient) AND variation (e.g. standard deviation) or associated estimates of uncertainty (e.g. confidence intervals)
☐	☒	For null hypothesis testing, the test statistic (e.g. *F*, *t*, *r*) with confidence intervals, effect sizes, degrees of freedom and *P* value noted<br>*Give P values as exact values whenever suitable.*
☒	☐	For Bayesian analysis, information on the choice of priors and Markov chain Monte Carlo settings
☒	☐	For hierarchical and complex designs, identification of the appropriate level for tests and full reporting of outcomes
☒	☐	Estimates of effect sizes (e.g. Cohen's *d*, Pearson's *r*), indicating how they were calculated

Our web collection on statistics for biologists contains articles on many of the points above.

Software and code

Policy information about availability of computer code

Data collection	All data collection was done through Python 3; For computational studies specifically, we used Pytorch v0.3; custom codes used in this study are available on GitHub repository (https://github.com/KangByungwoo/occlusion_public_repo).
Data analysis	All data analysis was done through Python 3; For computational studies specifically, we used Pytorch v0.3; custom codes used in this study are available on GitHub repository (https://github.com/KangByungwoo/occlusion_public_repo).

For manuscripts utilizing custom algorithms or software that are central to the research but not yet described in published literature, software must be made available to editors and reviewers. We strongly encourage code deposition in a community repository (e.g. GitHub). See the Nature Portfolio guidelines for submitting code & software for further information.

Data

Policy information about availability of data

All manuscripts must include a data availability statement. This statement should provide the following information, where applicable:

- Accession codes, unique identifiers, or web links for publicly available datasets
- A description of any restrictions on data availability
- For clinical datasets or third party data, please ensure that the statement adheres to our policy

Datasets used in the study is available on GitHub repository (https://github.com/KangByungwoo/occlusion_public_repo).

Research involving human participants, their data, or biological material

Policy information about studies with human participants or human data. See also policy information about sex, gender (identity/presentation), and sexual orientation and race, ethnicity and racism.

Reporting on sex and gender	Sex and/or gender were not relevant to the study design, and no sex- or gender-based analyses were performed.
Reporting on race, ethnicity, or other socially relevant groupings	Race, ethnicity, or other socially relevant groupings were not considered in the study design.
Population characteristics	Basic demographic information was collected (age, race, sex), but it wasn't part of the study design and wasn't a feature in any of the analyses. Participants were English-speaking adults residing in the U.S. (mean age = 37.7 ± 9.1 years, range 21–68; 68.6% male, 31.4% female). Their self-reported race/ethnicity is as follows: White/Caucasian 71.4%, Hispanic 8.6%, Black/ African American 8.6%, Indian 5.7%, East Asian 2.9%, Other 2.9%, Southeast Asian, Native Hawaiian or Pacific Islander, Native American, and Middle Eastern/North African 0%. See the Methods section for more details.
Recruitment	Participants were recruited online via Amazon Mechanical Turk. Participants were self-selected and may not represent the general population of U.S. adults. Potential self-selection bias could reflect differences in familiarity with online tasks or motivation related to monetary compensation. However, the task design minimized such effects by using within-subject comparisons, randomized trial presentation, and performance-based incentives.
Ethics oversight	Stanford University Institutional Review Board.

Note that full information on the approval of the study protocol must also be provided in the manuscript.

Field-specific reporting

Please select the one below that is the best fit for your research. If you are not sure, read the appropriate sections before making your selection.

☒ Life sciences ☐ Behavioural & social sciences ☐ Ecological, evolutionary & environmental sciences

For a reference copy of the document with all sections, see nature.com/documents/nr-reporting-summary-flat.pdf

Life sciences study design

All studies must disclose on these points even when the disclosure is negative.

Sample size	No formal statistical method was used to predetermine sample size. At least N = 5 samples were used per group, with sample numbers chosen to ensure robust statistical analyses within the limits of available computational resources.
Data exclusions	No data were excluded from the analyses.
Replication	All key findings were derived from aggregated results across multiple participants or experimental runs. Beyond replication across these independent samples, no additional replication experiments were performed.
Randomization	Randomization was not applicable, as the study design did not involve group allocation. All participants were exposed to the same experimental conditions.
Blinding	Blinding was not performed, as the nature of the study did not involve group comparisons or subjective outcome assessments that would necessitate blinding.

Reporting for specific materials, systems and methods

We require information from authors about some types of materials, experimental systems and methods used in many studies. Here, indicate whether each material, system or method listed is relevant to your study. If you are not sure if a list item applies to your research, read the appropriate section before selecting a response.

Materials & experimental systems

n/a	Involved in the study
☒	☐ Antibodies
☒	☐ Eukaryotic cell lines
☒	☐ Palaeontology and archaeology
☒	☐ Animals and other organisms
☒	☐ Clinical data
☒	☐ Dual use research of concern
☒	☐ Plants

Methods

n/a	Involved in the study
☒	☐ ChIP-seq
☒	☐ Flow cytometry
☒	☐ MRI-based neuroimaging

Plants

Seed stocks	*Report on the source of all seed stocks or other plant material used. If applicable, state the seed stock centre and catalogue number. If plant specimens were collected from the field, describe the collection location, date and sampling procedures.*
Novel plant genotypes	*Describe the methods by which all novel plant genotypes were produced. This includes those generated by transgenic approaches, gene editing, chemical/radiation-based mutagenesis and hybridization. For transgenic lines, describe the transformation method, the number of independent lines analyzed and the generation upon which experiments were performed. For gene-edited lines, describe the editor used, the endogenous sequence targeted for editing, the targeting guide RNA sequence (if applicable) and how the editor was applied.*
Authentication	*Describe any authentication procedures for each seed stock used or novel genotype generated. Describe any experiments used to assess the effect of a mutation and, where applicable, how potential secondary effects (e.g. second site T-DNA insertions, mosiacism, off-target gene editing) were examined.*
